# Parental perceptions and experiences of kangaroo care for preterm infants in neonatal intensive care units in China: a qualitative study

**DOI:** 10.1186/s12884-024-06622-9

**Published:** 2024-07-25

**Authors:** Qian Cai, Yunxian Zhou, Danqi Chen, Fang Wang, Xinfen Xu

**Affiliations:** 1 Women’s Hospital School of Medicine Zhejiang University, No.1 Xueshi Road, Shangcheng District, Hangzhou, Zhejiang Province 310006 China; 2 Department of Nursing, Zhejiang University School of Medicine, Hangzhou, Zhejiang China; 3 School of Nursing, Zhejiang Chinese Medical University, Hangzhou, Zhejiang China

**Keywords:** Experience, Kangaroo care, Neonatal intensive care unit, Parent, Perception, Premature, Qualitative research

## Abstract

**Background:**

kangaroo care (KC), endorsed by the World Health Organization, is an evidence-based intervention that plays a pivotal role in mitigating preterm infant mortality and morbidity. However, this intervention has not been fully integrated into healthcare systems in China. This study aimed to gain insight into parents’ perceptions and experiences of KC for preterm infants to contribute to the KC implementation on a larger scale.

**Methods:**

This study employed a descriptive qualitative design, using face-to-face, semi-structured, in-depth interviews. Fifteen parents participating in KC for preterm infants in the neonatal intensive care units (NICUs) were purposively sampled from four hospitals across four cities in Zhejiang Province, China. Thematic analysis was employed to analyze the data.

**Results:**

Four themes and twelve subthemes regarding the parents’ perceptions and experiences about KC were identified. The four themes included: (1) Low motivation upon initial engagement with KC, (2) Dynamic fluctuations of emotional states during KC, (3) Unexpected gains, and (4) Barriers to participation.

**Conclusions:**

Parents’ perceptions and experiences of KC was a staged process, with parents exhibiting distinct cognitive patterns and unique experiences at each stage. Overall, as KC progresses, parents’ experiences tended to become increasingly positive, despite potential obstacles encountered along the way. To enhance the implementation of KC, healthcare providers could utilize prenatal and postnatal education programs. These programs aim to enhance the understanding of KC among parents of preterm infants, fostering sustained engagement in KC practices.

## Background

According to the World Health Organization (WHO), an estimated 13.4 million preterm infants were born globally in 2020, representing more than one in ten of all births [1, 2]. Complications of preterm birth constituted the primary cause of mortality among children under five years old, resulting in approximately 900,000 deaths in 2019 [3]. Many surviving infants faced lifelong disabilities, including cognitive impairments and visual and auditory deficits [4–6]. In 2022, the WHO revised the preterm guidelines stating that kangaroo care (KC) is to be the norm for all preterm infants due to its effectiveness in enhancing the survival and well-being of preterm infants [7].

KC means placing a naked preterm infant prone on the bare chest of his/her caregiver (such as father or mother) in the same way as kangaroo parenting, facilitating early, continuous, and long-term skin-to-skin contact between the preterm infant and the caregiver, while measures such as exclusive breastfeeding or breastfeeding, early discharge, and follow-up after discharge are taken for the preterm infants [8–10]. Compared to conventional care, KC is not only able to reduce neonatal mortality by 37–40%, but also significantly lower the incidence of sepsis, hypoglycemia, and hypothermia [11, 12]. Numerous studies have demonstrated that KC is a safe, efficacious, and multifaceted intervention with many short-term and long-term positive effects for preterm infants [13–16]. Despite the well-established benefits of KC, this intervention has not been fully integrated into healthcare systems globally [17, 18].

The incidence of preterm birth in China is among the highest in the world and has been increasing year by year [2, 19]. Preterm birth complications remained a leading cause of neonatal mortality in China [20], indicating that clinical care for preterm infants was still insufficient [21]. Although KC programs have been piloted in some Chinese hospitals, KC has not been widely disseminated and implemented in China and was not yet routine care for preterm infants in the neonatal intensive care unit (NICU) [22, 23]. One of the main reasons is that most NICUs in China adopt restricted visiting policies in which parents are denied entry or visits are limited to a few minutes per week [24]. Therefore, most NICUs allow very limited parental access, making KC difficult to implement. Previous research by Cai et al. [25] demonstrated that factors influencing KC implementation mainly include environmental factors, professional factors, parent/family factors, access factors, and cultural factors. Among these, insufficient awareness and misconceptions about KC among parents/family members were identified as a significant barrier to the practice of KC [25]. Parents of preterm infants were the primary participants in KC implementation, and their knowledge and perceptions about KC directly influenced their decision-making and behaviours, including their willingness to accept KC and their level of engagement in the process [25, 26]. Another study has shown that due to the ignorance of KC, some parents felt worried that participating in KC may harm their preterm infants and might even be reluctant to have skin-to-skin contact with their preterm infants [27].

Overall, healthcare providers should be aware of the cognition level of preterm infants’ parents for KC and their experience of implementing KC to provide targeted support. Studies have demonstrated that the initial experience and views of preterm infants’ parents in participating in KC are crucial for promoting the implementation of KC [28, 29]. Sufficient training and education interventions can enhance the cognition level of preterm infants’ parents for KC in the NICU, and guidance from nurses and other clinical professionals can improve their experience [30]. However, due to the difference in visiting systems, resources and culture [24, 31], the outcomes from qualitative studies conducted abroad might not be readily transferable to the Chinese context. Moreover, previous qualitative research on parents’ perceptions and experiences with KC in NICUs in China was scarce. Only few single-center qualitative studies had been conducted which did not comprehensively capture parents’ views and perceptions on KC [32, 33].

The purpose of this study was to explore the perceptions and experiences of parents of preterm infants participating in KC in NICUs of four hospitals in Zhejiang Province, China, through a descriptive qualitative study. These findings could not only provide a basis for improvement for localizing KC program strategies implementation in China but also offer valuable references for other developing countries seeking to promote and apply KC in NICUs with restrictive visiting systems.

## Methods

### Study design

A qualitative descriptive approach was utilized to capture the perceptions and experiences of parents engaging in KC with their preterm infants in the NICU. This method is especially suitable where there is a need to understand a phenomenon in depth when little is known about it. Furthermore, the results produced in a qualitative descriptive study stay close to the data as given, ensuring that they are grounded in the actual experiences and words of the participants [34, 35]. Results were reported according to the consolidated criteria for reporting qualitative research (COREQ) guideline [36].

### Study setting

This study was conducted from January to April 2023 in NICUs of four tertiary hospitals in diverse cities (Hangzhou, Shaoxing, Jiaxing, and Huzhou) of Zhejiang Province, each hospital housing a single NICU, ensuring a diverse sample for our qualitative descriptive study on KC. Hospital selection was guided by their comparatively high overall KC utilization rates in their NICUs, while also considering convenience and accessibility for this study.

### Sampling and participants

Study participants were recruited with the assistance of designated KC research coordinators (typically the head nurses of the respective wards) in each hospital. Purposeful sampling was employed, adhering to the principle of maximum variation strategy to select participants with diverse characteristics [37], including age, education, occupation, income, gestational age and birth weight of preterm infants, to obtain a comprehensive sample source.

Inclusion criteria for preterm infants were as follows: (1) gestational age at birth within 37 weeks (<259 days), (2) NICU stay of ≥ 7 days with stable vital signs. Exclusion criteria were: (1) presence of congenital malformations or genetic disorders, (2) secondary admission to the NICU. Parents of preterm infants who met these criteria were included in the study. Additionally, parents of preterm infants were required to: (1) have participated in KC ≥ 3 times, (2) have no cognitive or communication barrier, (3) have provided informed consent and voluntarily participated. In cases where both parents had participated in KC, they were asked to designate one parent to be interviewed. Parents of preterm infants with a history of psychiatric or mental disorders were excluded.

### Data collection

The data was collected through individual face-to-face, semi-structured interviews at each of the four healthcare facilities, and every interview lasted between 35 and 60 min. Semi-structured interviews were utilized to enable the interviewer to probe issues that may be of interest to the current research but are not explicitly addressed by the interview guide [37]. The initial interview outline was developed by the first author and revised based on a group discussion. A pilot test was conducted to refine the interview outline and develop relevant lines for questioning [38]. Specifically, before initiating the formal interviews, we carried out two pilot interviews in January 2023 within the NICUs of Hangzhou and Shaoxing, each pilot interview conducted at the respective hospital, to refine the interview guide to better capture the nuanced experiences and perceptions of the parents regarding KC. The final interview questions are shown in Table 1.

**Table 1:** Semi-structured interview guide

Interview questions
1. Could you share your perceptions and experiences with KC and in what ways has this experience affected you?
2. How did you feel about your first KC session? How have your feelings and perceptions changed after your first KC session?
3. What are your opinions on the KC program in the NICU?
4. What have you gained from participating in KC ? What other expectations do you have for participating in KC?
5. What concerns or difficulties do you have while participating in KC? What support or assistance have you received during this process ?
6. What questions do you have on KC for preterm infants? What other needs do you have?
7. What are your suggestions to help parents better participate in KC for preterm infants?

*Abbreviations* KC, kangaroo care; NICU, neonatal intensive care unit

Before the interviews, a calm and private setting (such as the head nurse’s separate offices) was arranged to offer a serene space shielded from the ambient noise and daily activities of the NICU, which not only prevented disruptions to the conversation but also fostered a sense of psychological safety for the participants, encouraging them to share their thoughts and feelings without reservation. All interviews were conducted in Chinese by the first author QC to ensure consistency. Before the interview began, a questionnaire, developed based on a literature review and team discussion, was distributed to each participant for collecting demographic details (age, education, occupation and so on). At the onset of each interview, the primary researcher introduced herself and provided a clear explanation of the topics and purposes of the interview to ensure that participants had a comprehensive understanding of the study before proceeding with the data collection process. Introductory questions (e.g. how are you feeling today?) were asked to establish rapport and put participants at ease. The interview guide steered the conversation, with additional probing based on the participants’ responses.

In-person interviews were audio-recorded with participants’ consent obtained before recording. Each parent was interviewed once, and field notes were taken by the interviewer. The pilot interviews conducted during the initial data collection period were not included in the data analysis. Data collection and analysis occurred concurrently. Saturation was deemed achieved when no further themes or concepts were identified from the interviews [39]. Specifically, after the 13th interview, there were no new themes generated from the interviews in our study. To ensure that we achieved data saturation, we proceeded with two additional interviews to ensure and confirm that no new themes were emerging. Accordingly, a total of 15 parents of preterm infants were interviewed in our study.

### Data analysis

All interview data were transcribed verbatim and carefully examined by the first two authors. The interview transcripts and audio files were then imported into NVivo 12 software (QSR International, Melbourne, Australia) for organizing and managing qualitative data. The thematic analysis, following Braun and Clarke’s approach [40], was guided by one research question: what experiences and perceptions influence parental engagement in the implementation of KC for preterm infants. The first and second authors (QC and YZ) independently read the initial four transcribed interviews multiple times and performed the initial coding. Subsequently, the first and second authors immersed themselves in the remaining 11 interview transcripts, generating initial sub-themes and themes using NVivo software. Then, the two authors reviewed the identified themes for internal homogeneity (i.e., meaningful cohesion of codes) and external heterogeneity (i.e., clear distinctions among themes) and the representation of the sub-themes and themes was refined by all members of the research team. Finally, the first author presented the data analysis results in a narrative format, after a consensus on clear definitions and names was reached on all sub-themes and themes. The second author YZ, a female qualitative research expert with a Ph.D. from Australia and multiple published qualitative studies, performed quality control throughout the study. Disagreements in coding and categorization were discussed promptly.

The data analysis was conducted in Chinese, and selected thematic codes were translated into English for presentation in the results section. To avoid any misinterpretation or inaccurate translation, a rigorous process involving forward translation, back-translation and reconciliation of discrepancies was employed in our study. The first author interviewer (QC), a female PhD candidate fluent in Chinese and English, performed the initial forward translation and the back-translation. Any differences that emerged during the back-translation were carefully reviewed and reconciled by the second author (YZ).

### Rigour

Rigour was achieved by focusing on four dimensions: credibility, transferability, dependability and confirmability [41, 42]. Credibility was addressed by engaging with the data extensively and engaging in thoughtful reflection throughout the analysis process. We maintained a reflexive audit trail, documenting each step of the analysis process, including the researcher’s reflective notes. Additionally, we conducted peer debriefing sessions to ensure thoughtful consideration of the data. To address transferability, purposive sampling was employed and provided comprehensive clinical, sociodemographic, and contextual information about the participants. The use of verbatim transcriptions, detailed field notes, and a record of analytical decisions contributed to the dependability of our study. Confirmability was established by incorporating illustrative data extracts in our findings.

#### Ethical considerations

This study received ethical approval from the Ethics Committee of the Women’s Hospital, School of Medicine, Zhejiang University (Approval No. IRB-20,220,219-R). Prior to their participation, written informed consent was obtained from all participants. They were fully informed about the study’s objectives and assured that their participation was voluntary. Participants were also informed of their right to withdraw from the study at any time without any negative consequences regarding the services they received.

Issues of concern included participant confidentiality and emotional well-being during the interviews. It was required to address sensitive topics which may cause a potential emotional impact on the participant and the researcher. Measures included providing contact information for emotional and mental health resource follow-up. Participants were clearly informed that they could stop the interview at any time if they experienced anxiety or distress, and they had the right to withdraw from the study at any time without any consequences. Confidentiality of their personal information was strictly maintained throughout the study. Specifically, participant demographic data, recorded interviews, and interview transcripts were all deidentified with a pseudonym to protect participants’ identity in our study.

## Results

### Participant characteristics

A total of 15 parents of preterm infants, including 12 mothers and three fathers, were interviewed in this study. The parents’ ages ranged from 26 to 40 years old, with a mean age of 30.73 years. Their educational levels varied from high school to university graduates. Among the infants of the 15 participants, seven were delivered via cesarean section and the others were delivered through vaginal birth. The infants were born between 24 + 5 and 34 + 5 weeks gestation, and birth weights ranged from 710 to 2270 g. All infants were hospitalized at the time of the interviews. Table 2 presents a summary of the participants’ demographic characteristics and clinical information. The analysis generated four themes and twelve subthemes, as shown in Table 3.

**Table 2:** Characteristics of participants (*N* = 15)

No.	Gender	Age (years)	Education level	Occupation status	Residential areas	Religion	Per capita monthly household income(Yuan)	Delivery mode	Infant’s gestational age (weeks)	Infant’s birth weight (g)	Infant’s LOS in hospital at the time of interview(days)	Withdraw or not before the discharge
P1	Female	31	College	Employed	Urban	No	10,001–15,000	C-section	31 + 0	1440	45	No
P2	Female	26	Bachelor	Employed	Rural	No	10,001–15,000	Vaginal delivery	29 + 5	1600	45	No
P3	Female	31	Senior high School	Unemployed	Rural	No	≤ 5000	Vaginal delivery	30 + 0	1770	61	No
P4	Female	31	Senior high School	Employed	Rural	No	5001–10,000	Vaginal delivery	25 + 0	800	70	No
P5	Female	27	College	Unemployed	Rural	No	15,001–20,000	Vaginal delivery	24 + 5	710	41	No
P6	Male	26	Senior high School	Freelancer	Rural	No	10,001–15,000	C-section	34 + 2	1970	27	Yes
P7	Female	37	Junior high school	Unemployed	Rural	No	≤ 5000	C-section	30 + 0	1000	50	Yes
P8	Female	40	Junior high school	Unemployed	Rural	No	5001–10,000	C-section	31 + 6	1800	16	Yes
P9	Female	30	Junior high school	Employed	Rural	Buddhism	5001–10,000	C-section	29 + 6	1090	47	No
P10	Female	32	Junior high school	Unemployed	Rural	No	≤ 5000	C-section	30 + 5	1210	39	No
P11	Male	31	College	Employed	Urban	No	5001–10,000	Vaginal delivery	34 + 5	2270	18	Yes
P12	Male	29	College	Freelancer	Urban	No	5001–10,000	C-section	32 + 3	1600	17	No
P13	Female	32	Bachelor	Employed	Urban	No	≥ 20,000	Vaginal delivery	30 + 6	1440	28	No
P14	Female	29	Bachelor	Employed	Rural	No	10,001–15,000	Vaginal delivery	30 + 0	1530	27	No
P15	Female	29	College	Employed	Urban	No	5001–10,000	Vaginal delivery	27 + 6	1210	56	No

*Abbreviations* LOS, length of stay

**Table 3:** Themes and subthemes

Themes	Subthemes
Low motivation upon initial engagement	Passive acceptance
with KC	Visitation opportunities
	Transition before discharge
Dynamic fluctuations of emotional states during KC	Eager to participate yet concerned about not being competent
	From nervous and fear to calm and confidence
	From unreassured to gratitude
Unexpected gains	Awakening parental roles
	Enhancing caregiving self-efficacy
	Emotion satisfaction of family members
Barriers to participation	Conflict between work/family affairs and KC
	Long commutes and parking inconvenience
	Financial constraints

*Abbreviations* KC, kangaroo care

### Theme 1: low motivation upon initial engagement with KC

Insufficient KC-related education during the prenatal period resulted in a marked knowledge deficit among parents of preterm infants regarding KC. Specifically, parents lacked an understanding of the clinical benefits of KC and harboured misconceptions about the motivations for participation. Consequently, parents exhibited low motivation upon initial engagement with KC, characterized by a lack of proactive participation.

### Passive acceptance

Preceding NICU admission, the majority of interviewed parents of preterm infants possessed limited knowledge of KC, with a subset reporting no prior exposure to the concept. Given that comprehension of KC’s clinical efficacy exerts a significant influence on parental engagement, parents did not articulate a pronounced desire or perceived need for KC upon being informed about it. Their participation was predominantly characterized by passivity, with adherence to medical recommendations being the primary motivator. The experience was frequently accompanied by feelings of perplexity and disorientation.

“I came here to do KC, but I only saw a sign when I got to the entrance. Honestly, I’m still not entirely clear what KC is. When the doctor told us that I could start KC, I had no idea what it was. We even asked a friend what the benefits of KC were.” (P7).

### Visitation opportunities

While certain parents of preterm infants possessed a degree of familiarity with KC, their comprehension of its clinical efficacy remained inadequate, leading them to prioritize KC primarily as a means of visitation. Postnatal admission of preterm infants to the NICU and subsequent visitation restrictions for parents in China result in prolonged separation. Superficial knowledge of KC prompted parents’ willingness to adopt this care model, with the desire to see and interact with their preterm infants serving as the most direct and strongest motivation for initiating KC.

“Normally, newborns cannot be visited in that department, it is a chance for me to see my baby. I just want to come and have a look at my baby.” (P6).

“I think most mothers probably still mainly want to see their babies. The biggest thing is to alleviate the longings and anxieties that have persisted for so long. I felt much more relieved and at ease after seeing my infant.” (P4).

### Transition before discharge

Some interviewees reported that they had learned from the experiences of other parents that doctors typically only recommended KC when the preterm infant’s condition was relatively stable and approaching discharge from the hospital. Consequently, they perceived KC as a harbinger of impending discharge and a straightforward transition before returning home. They believed that implementing KC signified the proximity to discharge and a resumption of family life.

“KC is typically initiated right before discharge, when the baby’s health has improved, allowing the mother to engage in KC. Therefore, when I heard about KC, I felt extremely excited.” (P15).

Despite a lack of comprehensive understanding and awareness of the clinical benefits of KC, interviewees expressed appreciation for the transitional phase it provided. They acknowledged that they might have been apprehensive about taking their preterm infant home directly, whereas KC provided emotional adaptation and transition for parents to care for their preterm infants at home. During KC implementation, they could familiarize themselves with their infants and gain experience in their care, thereby enhancing their preparedness for home care before discharge from the hospital.

“We are being parents for the first time with no prior experience, our baby went straight into the NICU right after birth, and we didn’t even get a chance to hold him. Having this transitional phase is valuable for us. It provides us with some understanding of how to care for him once we are back home.” (P14).

### Theme 2: dynamic fluctuations of emotional states during KC

Parents of preterm infants experienced a spectrum of emotions during their involvement in KC which fluctuated over time, following a progressive and evolutionary trajectory. Overall, the emotional experience was characterized by a gradual shift towards being positive. Furthermore, KC promoted the establishment of a positive and healthy healthcare provider-parent relationship, leading parents to express increased trust and gratitude towards healthcare professionals.

### Eager to participate yet concerned about not being competent

Since there was no opportunity to interact with their preterm infants, parents initially expressed enthusiasm and eagerness to participate in KC upon learning about its availability. However, this desire was primarily motivated by a longing to see their preterm infants rather than a desire to practice KC.

“When the doctor said that we could start KC the next day, I was so excited and really looking forward to seeing my baby.” (P10).

Meanwhile, parents experienced concerns and apprehensions about providing care for their preterm infants. They were apprehensive about their ability to ensure adequate care due to their lack of experience, and the potential for causing harm to their vulnerable infants during KC. This concern was particularly pronounced among fathers, who worried that their big and clumsy hands could inadvertently injure their fragile infants.

“When I saw those tiny hands and feet that day, I felt a bit worried. I was concerned that I might use too much force or make too sudden movements, afraid that I wouldn’t do it well.” (P12).

### From nervous and fear to calm and confidence

When parents first hold their preterm infants for KC, they experienced hesitation, apprehension and fear regarding even basic tasks. Some parents reported negative emotions when instructed to observe monitors and pay attention to changes in their infants’ vital signs. The infants’ diminutive size and the presence of medical tubes caused parents of preterm infants worried about their ability to provide adequate care.

“The first few times I did KC, my palms would sweat, and I felt very nervous. I didn’t know whether I was doing it right, everything was in a mess. Their tiny arms and legs, their bones were all soft, and I had no experience at all, so I didn’t dare to touch anything, I just didn’t know where to start.” (P14).

However, with an increasing number of KC sessions, parents reported a concurrent rise in their caregiving confidence. The initial apprehension and hesitancy gradually dissipated, and they began to feel calm and reassured internally. One reason for this change was the increasing practice and familiarity with the process, while another reason was the guidance and assistance provided by healthcare professionals.

“After a day or two of holding them, I gradually felt reassured. I felt like that everything was fine. As time went on, I no longer had to be so cautious and my mood became calm and steady as well.” (P9).

### From unreassured to gratitude

In the context of restricted visiting policies in Chinese NICUs, parents of preterm infants might have initially experienced a lack of understanding and trust in healthcare professionals. This stemmed from limited knowledge about their infants’ care, as they primarily relied on infrequent phone updates from healthcare professionals. Consequently, without direct observation of their infants’ care, parents might harbour concerns about the quality of care and the level of attention their infants were receiving, potentially leading to fears that their infants’ needs were not being timely and adequately met.

“If you only rely on the doctor’s weekly phone calls to know how the infants are being taken care of in the hospital without seeing it with your own eyes, then you definitely don’t know how the baby is being cared for, which would make us feel worried and uneasy.” (P5).

However, through participation in KC, some interviewees reported experiencing close contact with healthcare professionals. This immersive experience allowed them to witness firsthand the patience and meticulous care provided to their preterm infants, gradually instilling a sense of reassurance and trust in the healthcare team. Additionally, face-to-face communication with healthcare professionals enabled parents to perceive their dedication more clearly and intuitively, fostering a deep sense of gratitude.

“When I have close contact and communication with the healthcare professionals, I could know more about them… I can ask the nurse about my infant’s condition like how much milk he’s drinking, and the nurse patiently tells us, so we feel more reassured and very grateful to the nurses.” (P10).

### Theme 3: Unexpected gains

Despite the low awareness level of KC, parents experienced unexpected benefits after participating in the program. While their initial expectations were primarily focused on visiting their infants and ensuring their health and well-being, they discovered additional positive outcomes. These included a deeper understanding of their parental roles, enhanced self-efficacy in caregiving, and fulfilment of emotional needs within their families. While individual experiences and emotions varied, these unexpected benefits were consistently positive and meaningful, contributing to the overall well-being of both parents and their preterm infants.

### Awakening to parental roles

Initially, most participants perceived KC as a means to visit their infants. However, during their first KC session, the experience of holding their babies and engaging in skin-to-skin contact evoked a profound realization of their parental identity. Many reported noticing their infants’ emotional cues and experiencing a sense of reciprocal communication. This ignited a deep sense of fatherhood/motherhood, instilling within them a profound understanding of their parental roles and responsibilities.

“I felt like a mother for the first time when I held my baby. Since I hadn’t had much contact with infants before, I felt so happy when I touched him. When I held him like this, really, I really felt like he’s my baby.” (P15).

Embracing their parental identity heightened their awareness of the significance of their role in their infants’ lives. It fostered a sense of protectiveness and love, motivating them to invest more fully in their infants’ care. This, in turn, reduced the stress associated with their parental responsibilities and facilitated a smooth transition into their new roles. Furthermore, the sense of accomplishment derived from successfully caring for their preterm infants reinforced their parental identity. This transformation was mutually beneficial, positively impacting the health and development of the preterm infants, while also supporting the emotional and psychological strength of the parents.

“It’s been so many days since the infant was born, but I had no feelings before I saw him. Now that I see him, I feel like the burden on my shoulders has increased. I realise that I have to strive to take on the role of a father, and gradually shift my focus from work to the family. If I haven’t done KC, maybe entering this role would take longer.” (P11).

### Enhancing caregiving self-efficacy

During KC, some parents initially perceived it merely as a transitional phase toward discharge. However, healthcare professionals encouraged and guided parents to actively participate in their preterm infants’ basic caregiving tasks beyond skin-to-skin contact. These tasks included feeding, diaper changing, massaging, and recognizing and responding to their infants’ cues. Through KC, parents became actively involved in their infants’ daily care, acquiring knowledge and skills related to preterm infant care. These hands-on experiences significantly enhanced parents’ caregiving competence.

“The nurses are here with me, teaching me things, providing guidance on how to hold the baby, how to feed, and how to change diapers, I gradually used to adapt to these tasks. They also share useful tips with me.” (P3).

Although some parents initially were anxious and at a loss about their caregiving abilities, their increased participation in KC fostered a gradual development of competence and confidence in caring for their infants. They became increasingly confident in their ability to provide adequate care and support for their preterm infants after discharge. This positive change made them more psychologically prepared when their infants were discharged from the hospital.

“Witnessing the baby steadily progressing in a positive direction, I feel more confident in my ability to handle feeding and other tasks upon returning home. Things are improving incrementally. It’s as if I now have a stronger sense of reassurance with myself. When I go back home, I will say that I can take care of my baby well.” (P13).

#### Emotion satisfaction of family members

Some interviewees said that participating in KC would enhance the bond between couples, leading to a closer connection and increased satisfaction in their relationship. This was because participating in KC provided a space for both parents to be jointly involved and committed to the care of their preterm infant in the early stages of life. During the implementation of KC, they entered a new life challenge together as a unit, where shared topics and frequency of communication increased, which had a positive effect on the emotional connection between the new parents.

“Our communication with each other became closer and more frequent. After I did the KC, he would ask how the baby was doing. I would tell him what the nurse had taught me, how to take care of the infant, how the infant was that day, whether he was well-behaved, whether he had cried, and so on. He was also very happy to hear about these things.” (P1).

Moreover, KC fostered relationships among family members by creating opportunities for them to stay informed about the preterm infant’s progress. During the KC program, interviewees shared photos and videos with their families, and after the KC, they would narrate the experience to their family members, providing updates on the infant’s growth and development. This process helped to alleviate anxiety and negative emotions within the family members, fostering a sense of shared joy and support.

“When I was doing KC, I took photos and videos to send to the family group. Grandma, grandpa, everyone was quite happy. Seeing everyone at home happy made me happy too. They all hoped to know him.” (P2).

### Theme 4: Barriers to participation

The guidelines suggested that the implementation time of KC for preterm infants should be extended as much as possible, and intermittent KC should be adhered to daily as much as possible to ensure the frequency of KC participation [43, 44]. However, we found that although some parents participated at the beginning, they may not continue to do so, or they may have participated sporadically. In our study, we found that there were many barriers that reduced the acceptance and participation of parents in KC, mainly in terms of time, physical distance, and costs.

### Conflict between work/family affairs and KC

During the hospitalization of preterm infants, mothers and fathers often played multiple roles. In addition to being concerned about the changes in the condition of preterm infants, they often needed to balance work and family affairs. At the same time, the closed ward management of the NICU required KC to be carried out within a fixed period, which undoubtedly increased the rigid time requirements for parents of preterm infants to participate in KC. The interviewees expressed their hope to participate in KC to get closer to their infants, but they were unable to do so due to their busy schedules. When time conflicts were unavoidable, they had to give up the opportunity to participate in KC, especially fathers of preterm infants who had to shoulder the responsibility of earning money to support their families.

“I have an older child to take care of at home, and there is no one to help with looking after him. My husband must work, while my mother-in-law, at most, helps with cooking because she is older. Basically, I must take care of the child at home on my own, so I can’t afford to spare any time.” (P8).

“The hospital has fixed schedules, so if my husband wants to participate, he must adjust his schedule. Currently, most fathers need to work to earn money. Especially for us migrant workers, it is not convenient and friendly. Usually, only the mother comes, as the father doesn’t have the time.” (P10).

#### Long commutes and parking inconvenience

In addition to affair conflicts and time constraints, a significant number of participants highlighted that the distance to the hospital was quite long and taking public transportation was rather time-consuming. For those opting to drive, parking at the hospital presented a formidable obstacle. Consequently, travelling to the hospital to participate in KC emerged as yet another hurdle.

“Sometimes, parking may take a long time, and there is no other way. My home is far, really quite far. It may take me about an hour and 10 minutes to drive there. The most annoying part is when I arrive, I can’t find a parking spot. I would have to drive around in circles there (to find a parking spot).” (P4).

Moreover, some mothers of preterm infants mentioned that the extended commute imposed a physical strain on them. The constant back-and-forth between the hospital and home resulted in inadequate rest. In China, the longstanding tradition of postpartum confinement, known as ‘ZUO YUE ZI’ advises mothers to recuperate at home for a month post-delivery, minimizing outdoor activities. These mothers expressed concerns that commuting to the hospital for KC exacerbated physical stress and fatigue, potentially hindering their postpartum recovery process.

“Taking public transport is quite troublesome because I live quite far away. It isn’t easy to come for each KC because it will take me about 6 hours round trip by bus, and I have to transfer and change buses. After all, it is not good for my recovery.” (P9).

#### Financial constraints

Financial constraints emerged as another barrier to KC implementation. Some respondents highlighted the substantial expenses that they had already incurred in the early period in the NICU for preterm infants. KC, as supplementary care, imposed an additional financial burden on families, especially for those from out-of-town or rural areas. Due to a lack of understanding about the clinical effectiveness of KC among some interviewees, they expressed confusion regarding the need to pay extra for “holding their babies”. Consequently, the associated fees may lead some parents to do KC sporadically or even give up KC.

“If the family’s financial situation is not good, we may consider saving every bit we could. We can tolerate not having a chance to hold the baby until discharge. The main issue is that the cost of KC is not covered by the medical insurance…It is hard for me to understand why we have to pay to hold our baby. It is very frustrating and incomprehensible. Anyway, we will see the baby sooner or later, so saving a little money is the priority.” (P7).

## Discussion

Preterm infants’ parents often exhibited low initial motivation to participate in KC, stemming from a lack of comprehensive understanding of the clinical effectiveness of KC and potentially distorted or inaccurate perceptions regarding KC. Interestingly, this low initial motivation did not negatively impact the emotional experience of the preterm infants’ parents. As they continued to participate in KC over time, their emotional experiences eventually turned positive, despite fluctuations during the process. Notably, the initial low motivation to some extent paradoxically paved the way for a subsequent emotional reversal, as the contrast between their initial low motivation and the positive experiences they encountered during KC sessions created a significantly intensive emotional shift. In addition to experiencing positive emotions eventually, preterm infants’ parents also gained unexpected benefits through their commitment to KC. Despite the presence of participation barriers, the trajectory of low initial motivation to unexpected gains characterized the overall experience of preterm infants’ parents participating in KC.

In our study, we found that the majority of parents had a lack of awareness regarding the clinical effectiveness of KC. The strongest initial motivation for most parents to participate in KC was the desire to visit and interact with their preterm infants, which was consistent with previous research [29]. Several studies have demonstrated that most preterm infants’ parents had very limited information about KC before entering the NICU to start KC and some had not even heard of KC [45, 46]. Moreover, this limited information was mostly not acquired from healthcare professionals but rather from the internet or other parents who had similar experiences [47], which resulted in parents’ initial perception of KC being distorted or inaccurate. Perception influences motivation, which in turn affects individuals’ attitudes and responses to barriers [48]. Although the motivation for visiting, as an external drive, could initially motivate parents of preterm infants to engage in KC, it inherently lacked the sustained driving force characteristic of internal motivation [49, 50]. In our study, four parents of preterm infants attempted KC but did not persist with the practice until their preterm infants were discharged. Previous study has described that parents were more likely to withdraw or even give up if they encountered obstacles during their participation in KC [51].

As indicated in our study, preterm infants’ parents often faced subjective barriers to KC participation, such as time constraints, long commutes, and financial burdens. When parents perceived KC solely as an opportunity for visitation rather than recognizing its clinical benefits, they might rationalize discontinuing participation upon encountering these challenges, which was confirmed in a systematic review encompassing 103 studies worldwide on the barriers and facilitators of KC practice [52]. In our study, we found that some parents experienced a decline in motivation or adherence to KC after initial visits due to their reluctance to bear the costs of extended visits and their inadequate understanding of KC, which hindered them from finding intrinsic motivation for KC.

Previous studies corroborated this perspective that low parental cognition about KC negatively impacted its acceptance and implementation [25, 26]. With low health literacy in China currently, it is crucial to leverage parents’ extrinsic motivation for visitation to initially engage them in KC. During the initial engagement period, healthcare providers should provide timely health education, emphasizing the clinical benefits of KC for preterm infants’ growth, development, and neurobehavioral outcomes [53]. This intervention is aimed at transforming extrinsic motivation into intrinsic motivation, thus fostering sustained participation [49]. However, the restrictive visiting system in China has resulted in relatively weak communication capacity among NICU healthcare providers [24]. They mainly guided parents on how to perform the KC, with insufficient emphasis on the clinical significance of KC, which should raise concern for healthcare providers in future. Therefore, healthcare professionals should provide educational programs for the parents of preterm infants in the future to enhance their awareness regarding the clinical advantages of KC.

Moreover, it is recommended to organize preterm infant parents who experienced the benefits of sustained KC participation to share their own successful experiences with parents who are about to start or have just begun KC [54]. According to Bandura’s social learning theory [55], people can learn certain social attitudes and behaviours by observing others or imitating role models. The role model effect and personal testimonies of previous preterm infant parents could be more persuasive and inspiring than health education provided by healthcare professionals. Studies have shown that peer support was a positive factor in promoting parental acceptance of KC implementation [25, 54]. Therefore, it is worth trying to leverage web-based support, such as the WeChat group (a Chinese social media platform for group dialogue), to form peer-to-peer support networks for parents of preterm infants [56, 57].

Apart from educating preterm infant parents about the clinical benefits of KC through the above-mentioned channels, the more important thing is to allow them to truly experience the changes and effects that KC brings to their participation. In our study, we found that over time, many parents progressed from initially low motivation to gradually experiencing the unexpected benefits of KC as they persisted in the process. For healthcare professionals, it is also important to communicate with preterm infant parents during this process, reinforcing their understanding of KC itself and the series of positive feelings and positive experiences that arise from practising KC [58]. Through this positive feedback, parents could continuously be aware of the benefits of participating in KC, thereby enhancing their willingness to continue KC. Some studies have also shown that the more preterm infant parents benefited from KC, the more likely they were to persevere [25, 27]. Therefore, healthcare professionals should pay more attention to parents who have not yet experienced the benefits of KC or have only seen minimal benefits during their participation.

In our study, some parents regarded KC as a transition before discharge. Although they did not fully understand or appreciate the clinical benefits of KC, they still attached great importance to it. Due to the restricted visiting policies in Chinese NICUs, preterm infants often required isolation and treatment in the NICU for several weeks to several months after birth, during which time parents had limited opportunities to enter the NICU to learn how to care for their infants [24]. KC provided an important opportunity for parents to initiate care for their infants in this situation, enabling them to transition into their parental roles more quickly [30]. Therefore, KC laid the foundation for intimate bonding between parents and their preterm infants, which was validated in previous studies [13, 59].

However, our study also revealed that parents initially had low self-efficacy and expectations of success in caring for preterm infants, and they generally felt uneasy, anxious and not confident of themselves. With increasing KC sessions, under the guidance and support of healthcare professionals, parents gradually acquired knowledge and skills in caring for preterm infants and their confidence in caring abilities was enhanced, which was consistent with previous study [28]. In other words, after surpassing the initial stage of low self-efficacy, positive self-efficacy feedback spontaneously formed as KC progressed, enabling them to participate in the care of preterm infants more actively. We could identify and leverage the emotional lows experienced by parents during this KC process, using the opportunity to teach caregiving skills to continually strengthen their confidence and provide targeted reinforcement for their self-efficacy. Parmar et al. [60] investigated the 7-day experience of 60 mothers and 40 fathers engaged in KC in India and reported that their confidence in parenting skills increased. This finding has also been corroborated by Moore et al. [61], which showed that mothers who provided KC exhibited less anxiety and more confidence in their ability to care for their infants after discharge.

Among the barriers hindering preterm infant parents’ acceptance and participation in KC, role conflict was a significant one. The birth of a preterm infant imposed a new life role for parents on top of their existing ones, which could lead to role conflict according to role conflict theory [62], as individuals were required to fulfil multiple roles in their social environment. This role conflict was particularly pronounced among fathers of preterm infants, as was shown in a previous study [63]. In our study, some preterm infant fathers chose to work instead of taking leave to care for their preterm infants due to the burden of financial pressure. They believed that working hard to earn money was an important way to fulfil their family responsibilities, especially considering the significant expenses incurred during the early hospitalization of the preterm infant [64]. This implies that alleviating the economic burden placed on the families of preterm infants is of great importance for resolving role conflict.

In the future, we could advocate for the inclusion of KC in medical insurance to alleviate the economic burden on the parents of preterm infants to a certain extent [57], to better support and promote the participation of fathers of preterm infants in KC. At the same time, hospitals may provide some flexibility in terms of time for parents participating in KC, allowing parents to make flexible appointments to practice KC [65], which could alleviate the time conflict. In a word, it is crucial to formulate targeted support strategies from the perspective of the parents of preterm infants to promote the implementation of KC in a way that is appropriate to local conditions, so as to scale up the national KC program.

### Limitations

The study had several limitations. First, the number of fathers who participated in this study was very low, since fathers are not encouraged to practice KC in Chinese culture and they are required to assume the responsibility of earning money to support their families. Second, all hospitals included in the study are tertiary hospitals, including general teaching hospitals and maternal and child healthcare hospitals located in major urban cities in Zhejiang. The experience and practice of KC in lower-level healthcare facilities may be different.

## Conclusions

Our study revealed that parents’ perceptions and experiences of KC was a staged process, with parents exhibiting distinct cognitive patterns and unique experiences at each stage. Overall, as KC progresses, parents’ experiences tended to become increasingly positive, despite potential obstacles encountered along the way. It is suggested that healthcare providers should leverage prenatal and postnatal education programs to enhance the understanding of KC among parents of preterm infants, fostering sustained engagement in KC practices. NICUs should establish supportive policies and programs approved by the hospital to address potential barriers and foster a conducive environment for KC practice, so as to facilitate parents’ ongoing involvement in KC. In future research, we may explore further insights from parents reluctant to KC as well as from staff to identify additional underlying factors obstructing the application of KC.

## Data Availability

The datasets used and/or analysed during the current study are in Chinese language and are not publicly available due to the confidentiality of the participants but are available from the corresponding author on reasonable request.
